# The myofascial release as neuromotor support to improve the ineffective sucking ability in term infants: a preliminary study

**DOI:** 10.1186/s13052-024-01611-2

**Published:** 2024-04-05

**Authors:** Andrea Arcusio, Maria Cristina Villa, Federica Felloni, Claudio Migliori

**Affiliations:** 1 Department of Rehabilitation Medicine, Ospedale San Giuseppe MultiMedica, Milan, Italy; 2 Department of Neonatology, Ospedale San Giuseppe MultiMedica, Milan, Italy

**Keywords:** Manual assessment, Neonate, Breastfeeding, Myofascial release, Sucking-swallowing, Osteopathy

## Abstract

**Background:**

Breastfeeding plays a primary role in the events that characterize the development of the relationship between a mother and her newborn. However, this essential process sometimes does not fully cover the nutritional requirements of the newborn due to altered biomechanical sucking skills. In this context, adequate osteopathic treatment associated with neuromotor facilitation techniques could play a promoting role.

**Methods:**

This study evaluated the effect of the osteopathic approach using myofascial release on 26 infants with ineffective sucking ability, identified by the POFRAS scale and LATCH score, compared with 26 untreated similar infants. After the procedure was initially performed in the hospital, the strategy based on basic neuromotor patterns was taught to the parents to be continued at home. The effects were measured at hospital discharge, during the first outpatient visit, which occurred after about seven days, and at one month of life.

**Results:**

The number of valid and continuous suctions, initially less than five per feed in both groups, at the first outpatient check-up was significantly higher (*p* < 0.00001) in the treated group. Exclusive breastfeeding, initially present in all enrolled children, was maintained mainly in treated children, both at discharge (*p* < 0.003), at outpatient follow-up (*p* < 0.00001), and at one month of life (*p* < 0.00001). Differences in growth and health conditions were not found between the groups.

**Conclusion:**

We believe that osteopathic treatment associated with neuromotor facilitation techniques can optimize newborns’ sucking skills, improving the acquisition and duration of breastfeeding.

## Background

Exclusive breastfeeding is considered the “gold standard” for newborns, as it provides optimal caloric intake, increases immunological protection, reduces the risk of metabolic pathologies both in the medium and long term, and has a positive effect on the mother-child relationship. Therefore, the ideal exclusive breastfeeding length should not be less than six months [1]. However, it is not rare for some women to interrupt breastfeeding already in the first month, and this is sometimes due to a biomechanical criticality of the newborn’s sucking-swallowing ability [2, 3]. Sucking is a neuromotor pattern that is correctly triggered if the somatic median line is stable, and this occurs through the activation of specific basic motor patterns [4, 5]. Breastfeeding requires coordination between sucking and swallowing, which are performed by the IX, X, and XII cranial nerves that control the efferent motor pathways of the tongue and orofacial muscles. Osteopathic manual treatment (OMT), using myofascial release techniques, aims to normalize musculoskeletal tensions of the neurotissue areas involved in the sucking-swallowing process [6]. Neuromotor facilitations (NF) are utilized in both healthy and pathological full-term neonates to activate basic skills [7, 8]. By training the basic neuromotor patterns, the orofacial muscles can be activated, which facilitates the motor system related to breast attachment and, therefore, breastfeeding [9].

The integration of OMT and NF in the rehabilitative management of altered somatic functions is already well-established and widely practised. The application of this combination in the neonatal field is especially intriguing and innovative.

The aim of the study was to verify the effects of the combination of OMT and NF on the sucking-swallowing process, in order to improve the acquisition and duration of breastfeeding. A group of newborns with low suction ability detected within the first 24 h of life was analyzed and compared to a similar control group.

## Methods

The study was conducted between April 2021 and September 2022 in the Neonatology department of San Giuseppe Multimedica Hospital. Written informed consent was obtained from the parents of each infant, and the study protocol was authorized by the local Ethics Committee.

To verify the effects of osteopathic manoeuvres on the sucking-swallowing process, and on the change in the percentage of maintaining exclusive breastfeeding in the first month of life, 350 newborns should have been enrolled. In order to reduce the risk of bias by involving a single osteopath specialist, with a large expertise in pediatric field for both evaluation and treatment, we chose to limit the duration of the study to 18 months. Consequently, the population analysed was found to be approximately 50 newborns.

Full-term neonates, regardless of the type of delivery, without any underlying medical conditions and in good general health were evaluated within the first 24 h of life to determine their appropriate sucking competence. This was assessed using the POFRAS scale [10, 11] and the LATCH score [12]. Infants who were unable to perform at least five valid and continuous suckles per feed, with persistent sleepiness and inadequate nutritional activity, were eligible for the study. Preterm infants, those with evident malformations or with a prenatal diagnosis of any pathology, and those who were small for gestational age or had intrauterine growth retardation were excluded. Newborns fed immediately with formula and those with sucking difficulties due to maternal anatomical problems (such as inverted or flat nipples) were also excluded, as well as infants whose parents refused to consent to the study. The parents of all the newborns enrolled in the study had attended childbirth preparation courses held by the obstetric and neonatology staff of San Giuseppe hospital. During these courses, ample information was provided for the promotion of breastfeeding in accordance with WHO recommendations.

The osteopathic evaluation identified the anatomical and physiological structures related to neonatal suction competence. Subsequently, myofascial release, a type of manual therapy that involves stretching the muscle-fascial complex with low load and long duration, was applied with the aim of improving its function [13]. The OMT treatment followed the standard techniques widely described in the literature [14, 15], choosing the method reported by Magoun [16] for neonatal treatment and applying it until the release of myofascial tension was achieved.

Neuromotor facilitations (NF) are the result of activation of basic neuromotor patterns, such as rolling, related to sucking activity.

Finally, parents were instructed in appropriate manoeuvres for their child’s specific neuromotor patterns, enabling them to continue them at home.

These procedures were carried out on the mother’s bed placed in the hospital room, following the “rooming-in” practice used at San Giuseppe Multimedica Hospital. Parents were instructed for half an hour each day, over two to three days during hospitalisation, with the osteopath directly observing their abilities in managing the neonates. Parents applied NF after every breastfeeding session during the hospital stay. Following discharge, the procedure continued at home until the first outpatient visit (approximately after seven days).

This allowed parents to learn and carry out the procedure in the same home conditions, coinciding with daytime feeding times and excluding the night period (from 11.00 pm to 6.00 am).

Several controls were scheduled to evaluate the effectiveness of the treatment: at hospital discharge (about 48–76 h after birth), after around 7 days during the routine outpatient visit, and at one month of life. Valid and continuous suctions, weight gain, and type of feeding (exclusive breastfeeding, bottle-fed only, or mixed) were used as indicators.

Statistical analysis was performed using either the two-tailed T-Student test to evaluate the average data of the groups or Friedman’s ANOVA for repeated measures to calculate and compare the variability of diet type at one month of life. A p-value greater than 0.05 was considered not significant.

## Results

Fifty-two newborns with a mean gestational age (± SD) of 38.6 ± 1.2 weeks and a mean birth weight (± SD) of 3,193 ± 391 gr were enrolled in the study. The infants were consecutively and alternately distributed into either the treated or control group, immediately after obtaining parental consent. One neonate in the control group did not complete the study, leaving a total of 51 babies, with 26 in the treated group and 25 in the control group, who were evaluated. There were no significant differences between the groups in terms of gestational age, sex, type of birth, birth weight, discharge weight, or the number of valid and continuous suckles at study entry (Table 1). All babies were initially exclusively breastfed. At the first outpatient evaluation, the number of valid suckling in the treated group was significantly higher compared to the control group (*p* < 0.00001). The number of infants who maintained exclusive breastfeeding was also significantly higher in the treated group (Table 2), beginning at hospital discharge (*p* < 0.003), and continuing to increase at the first follow-up evaluation (*p* < 0.001) and at one month of age (*p* < 0.00001). No significant difference in weight trends was found between the two groups.

**Table 1:** Patient characteristics

	Number of patients	Mean GA (wks.)	Type of birth	Gender	Mean BW (gr.)	Mean DW (gr.)	Continuous suction nr. in the first 24 h
**All babies**	51	38,6	CS = 33 VD = 18	F = 30 M = 21	3192	2959	3,62
**Treated**	26	38,7	CS = 18 VD = 8	F = 15 M = 11	3215	2999	4,08
**Controls**	25	38,5	CS = 15 VD = 10	F = 15 M = 10	3169	2914	3,72
***p***	NS	NS	NS	NS	NS	NS	NS

GA = gestational age; BW = birth weight; DW = weight at discharge, CS = caesarean section; VD = vaginal delivery; M = male; F = female

**Table 2:** Evolution of feeding during the study

		BM	MF	AM
**Treated**	*Discharge*	17	9	0
	*First control*	16	9	1
	*One month*	15	10	1
**Controls**	*Discharge*	10	7	8
	*First control*	7	7	11
	*One month*	4	5	16
**Statistical analysis**	*Discharge*	*p* < 0,003		
	*First control*	*p* < 0,001		
	*One month*	*p* < 0,00001		

BM = breast milk; MF = mixed feeding; AM = artificial milk

## Discussion

Osteopathy is a complementary branch of medicine that uses manual manipulation to treat somatic dysfunctions with the goal of improving patient health. The literature reports positive effects of osteopathy on neonates, including somatic dysfunctions [17] and reducing hospitalization in the NICU [18], proposing standardized methods for their evaluation and management [19]. Girgin [20] describes the importance of suction and swallowing motor patterns in the preterm population, while Azuma [21] extends the relationship between functional anomalies and nutritional difficulties to full-term neonates. Several studies report the efficacy of OMT in improving infants’ sucking-swallowing skills [22–24]. However, to our knowledge, the application of NF to basic motor patterns has never been involved. This study is the first to combine OMT and NF and involves parents in training their child’s neuromotor patterns, improving their skills and the mother-child relationship.

OMT, through myofascial release, can normalize musculoskeletal tensions. The association with neuromotor facilitations allows for a faster activation of neuromuscular coordination schemes aimed at sucking and swallowing.

Overall, our study found positive results in both the quantitative development of the suck-swallow process and the prolonged maintenance of exclusive breastfeeding up to one month of age. The observation until the first month of life serves as a compromise between maintaining the stability of the obtained results and minimizing potential biases. However, we believe that the association of OMT and NF directly affected only the suck-swallow process, while the prolonged maintenance of breastfeeding may be a consequence of the improvement in sucking ability. The increase in the LATCH score, a parameter utilized to measure sucking ability and known to be predictive of breastfeeding, observed as early as 24 h after the start of treatment, could explain the prolonged maintenance of breastfeeding as well as the continuation of OMT and NF performed by parents at home.

Although our study had a limited sample size, it suggests that osteopathic manual treatment associated with neuromotor facilitation could be a valuable approach to improve orofacial muscle efficiency and facilitate the suck-swallow process in infants with initially ineffective sucking skills. The inclusion of parents in the treatment process and their training in performing OMT and NF at home could also promote the duration of exclusive breastfeeding. Further studies involving a larger population of infants are needed to confirm our findings on the effectiveness of OMT and NF in improving neonatal suckling ability and breastfeeding maintenance. We hypothesize that the combination of OMT and NF could prove effective in improving the nutritional capabilities of premature infants, and especially in late preterm infants, frequently affected by ineffective sucking ability.

## Conclusion

In conclusion, our study suggests that OMT associated with NF could be a beneficial intervention for infants with ineffective sucking skills. The treatment improved the suck-swallow process, as demonstrated by the increase in LATCH scores and the number of infants who maintained exclusive breastfeeding up to one month of age. Moreover, involving parents in the home continuation of treatment could promote the duration of exclusive breastfeeding. However, our study had a small sample size, but the resulting Number Needed to Treat, approximately 2.5, encourages us to contemplate expanding the use of osteopathic manoeuvres in a larger population of newborns. Moreover further studies are needed to confirm our findings and to explore the long-term effects of the intervention on infant development. Overall, our study highlights the potential of complementary medicine in improving neonatal outcomes and promoting maternal-infant bonding.

## Data Availability

The datasets used and analysed during the current study are available from the corresponding author on reasonable request.

## Abbreviations

POFRAS: Preterm Oral Feeding Readiness Assessment Scale
LATCH: Latch, audible swallowing, type of nipple, comfort, hold
OMT: Osteopathic manual treatment
NF: Neuromotor facilitations
WHO: World health organization
ANOVA: analysis of variance
NICU: Neonatal intensive care unit
